# Generating survival times to simulate Cox proportional hazards models with time-varying covariates

**DOI:** 10.1002/sim.5452

**Published:** 2012-07-04

**Authors:** Peter C Austin

**Affiliations:** a Institute for Clinical Evaluative SciencesToronto, Ontario, Canada; b Department of Health Management, Policy and Evaluation, University of TorontoCanada; c Dalla Lana School of Public Health, University of TorontoCanada

**Keywords:** survival analysis, proportional hazards model, simulations, time-varying covariates, power and sample size calculation, time-dependent covariate, exponential distribution, Weibull distribution, Gompertz distribution

## Abstract

Simulations and Monte Carlo methods serve an important role in modern statistical research. They allow for an examination of the performance of statistical procedures in settings in which analytic and mathematical derivations may not be feasible. A key element in any statistical simulation is the existence of an appropriate data-generating process: one must be able to simulate data from a specified statistical model. We describe data-generating processes for the Cox proportional hazards model with time-varying covariates when event times follow an exponential, Weibull, or Gompertz distribution. We consider three types of time-varying covariates: first, a dichotomous time-varying covariate that can change at most once from untreated to treated (e.g., organ transplant); second, a continuous time-varying covariate such as cumulative exposure at a constant dose to radiation or to a pharmaceutical agent used for a chronic condition; third, a dichotomous time-varying covariate with a subject being able to move repeatedly between treatment states (e.g., current compliance or use of a medication). In each setting, we derive closed-form expressions that allow one to simulate survival times so that survival times are related to a vector of fixed or time-invariant covariates and to a single time-varying covariate. We illustrate the utility of our closed-form expressions for simulating event times by using Monte Carlo simulations to estimate the statistical power to detect as statistically significant the effect of different types of binary time-varying covariates. This is compared with the statistical power to detect as statistically significant a binary time-invariant covariate. Copyright © 2012 John Wiley & Sons, Ltd.

## 1. Introduction

Simulations and Monte Carlo methods serve an important role in modern statistical research. They allow for an examination of the performance of statistical methods in settings in which analytic and mathematical derivations may not be feasible. A key element in any statistical simulation is the existence of an appropriate data-generating process: one must be able to simulate data from an underlying statistical model.

Time-to-event outcomes occur frequently in the biomedical literature. In the medical literature, the Cox proportional hazards regression model is the most common approach for examining the effect of explanatory variables on time-to-event outcomes. Using this model, one is modeling the effect of explanatory variables on the hazard of the outcome. Prior studies have described methods to simulate data from a Cox proportional hazards model [1, 2]. Use of these data-generating processes allows for the examination of the performance of the Cox proportional hazards regression model in different settings.

Two advantages of the Cox proportional hazards regression model are its abilities to incorporate time-varying covariate effects and time-varying covariates [3, 4]. The former refers to a variable that is measured at baseline and whose value remains fixed over the duration of follow-up; however, the effect of this variable on the hazard of the outcome is allowed to change over the duration of follow-up. The latter refers to a variable whose value itself changes over the duration of follow-up. Examples of time-varying covariates in biomedical research include the receipt of an organ transplant, cumulative dosage of radiation or of a pharmaceutical agent, and compliance or adherence with a medication intended for chronic use. In the first example, receipt of an organ transplant is a dichotomous exposure or treatment. Subjects may change their exposure status from unexposed to exposed at most once during the follow-up interval. Once exposed, a subject remains exposed for the duration of follow-up. In the second example, cumulative dosage of radiation or to a pharmaceutical agent is a continuous time-varying covariate, whose value is nondecreasing over time. In the third example, current medication use also represents a dichotomous exposure. However, subjects may move both from unexposed to exposed and from exposed to unexposed during the course of follow-up. Thus, subjects may both initiate and discontinue treatment, and this pattern may be repeated during the course of follow-up. Throughout the remainder of the manuscript, we focus on simulating data in the presence of time-varying covariates and do not consider time-varying covariate effects. Correctly accounting for time-varying covariates is important because it allows one to avoid the issue of survivor-treatment or immortal-time bias [5–8]. Given a cohort study in which treatment or exposure occurs at some point during follow-up, this bias can occur when the analyst treats the exposure as being known and fixed at baseline. In so doing, the time until the application of the exposure is termed ‘immortal-time’, because by definition the exposed subject could not have died prior to the application of the exposure. Beyersmann *et al.* demonstrated that the biased hazard ratio will always be less than the true hazard ratio [6].

To conduct simulations of the performance of different statistical methods for use in settings with time-varying covariates, there is a need to describe data-generating processes for the Cox proportional hazard model in the presence of time-varying covariates. The paper is structured as follows. In Section 2, we present previous work on generating survival times to simulate Cox proportional hazards models with fixed or time-invariant covariates. These are covariates whose values are fixed at baseline and which do not subsequently change over the duration of follow-up. In Section 3, we extend these results to settings in which there is a time-varying covariate. We consider the case of the Cox-exponential model, the Cox–Weibull model, and the Cox–Gompertz model. In Section 4, we present an application of these methods to investigate the statistical power to detect as statistically significant the effect of different types of time-varying covariates on the hazard of an outcome. Finally, in Section 5 we summarize our findings and place them in the context of the existing literature.

## 2. Background

Let *h*(*t* | *x*) = *h*_0_(*t*) exp(*β* ′ *x*)denote the conventional Cox proportional model with fixed or time-invariant covariates, where *t* denotes time, *x* is the vector of time-invariant covariates, *β* is the vector of regression coefficients, and *h*_0_(*t*) is the baseline hazard function (the hazard function of the outcome occurring for those subjects with *x*= 0). The model describes the effect of the covariates on the hazard of the occurrence of the outcome.

The survival function of the above model is *S*(*t* | *x*) = exp( − *H*_0_(*t*) exp(*β* ′ *x*)), where *H*_0_(*t*) is the cumulative baseline hazard function, which is defined as 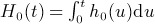. The distribution function of the event times under the Cox proportional hazards model is *F*(*t* | *x*) = 1 − exp( − *H*_0_(*t*) exp(*β* ′ *x*)). Both Leemis and Bender *et al.* have demonstrated that a survival time, *T*, can be generated by 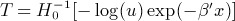, where *u* ∼ *U*(0,1) (where *U*(0,1) denotes the standard uniform distribution) [1, 2]. Simulating survival or event times from a Cox model with time-invariant covariates requires inverting the cumulative hazard function.

As noted by Bender *et al.*, among the commonly used distributions for survival times, only the exponential, the Weibull, and the Gompertz distributions also share the assumption of proportional hazards with the Cox model. The parameters required for each distribution, the hazard function, the cumulative hazard function, the inverse of the cumulative hazard function, and the formula for simulating survival times from each distribution in the setting of time-invariant covariates are described in Table I (see Ref. [2] for further details). Although there are several different parameterizations of the Weibull distribution, we use the parameterization of Bender *et al.*

**Table I:** Characterization of the exponential, Weibull, and Gompertz distributions

Characteristic	Exponential distribution	Weibull distribution	Gompertz distribution
Parameter	Scale parameter	Scale parameter *λ* > 0	Scale parameter *λ* > 0
	*λ* > 0	Shape parameter *ν* > 0	Shape parameter – ∞ < *α* < ∞
Hazard function	*h*_0_(*t*) = *λ*	*h*_0_(*t*) = *λνt*^*ν* − 1^	*h*_0_(*t*) = *λ*exp(*αt*)
Cumulative hazard function	*H*_0_(*t*) = *λt*	*H*_0_(*t*) = *λt*^*ν*^	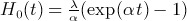
Inverse cumulative hazard			
function	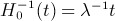	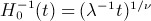	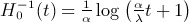
Simulating survival			
times (*u* ∼ *U*(0,1))	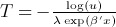	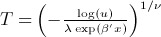	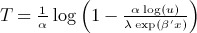

## 3. Generating survival times to simulate Cox proportional hazards models with time-varying covariates

In Section 2, we described the three commonly-used distributions that also satisfy the proportional hazards assumptions and previous work that described how one could simulate event times from these distributions when covariates were time-invariant. In this section, we extend these results to settings in which there is a time-varying covariate. We assume that there is a single time-varying covariate, which we denote by *z*(*t*), while we assume that the other covariates, *x*, are time-invariant. Furthermore, we let *β* denote the vector of regression coefficients associated with the vector of fixed covariates *x*, while *β*_*t*_ is the regression coefficient associated with *z*(*t*). We also assume that the logarithmic link function is used to relate the hazard function to the linear predictor: *h*(*t* | *x*(*t*)) = *h*_0_(*t*) exp(*β*_*t*_*z*(*t*) + *β* ′ *x*). Then, the cumulative hazard function is given by: 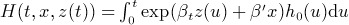.

We consider three different types of time-varying covariates: the first is a dichotomous time-varying covariate that can change at most once from untreated to treated (e.g., organ transplant); the second is a continuous time-varying covariate such as cumulative exposure to a fixed dose of radiation or a pharmaceutical agent; the third is a dichotomous time-varying covariate with a subject being able to move repeatedly from untreated to treated and back to untreated. We examine each of these types of time-varying covariates in subsequent sections. We present detailed derivations for the setting in which event times follow a Weibull distribution. Derivations for the other two distributions are presented in detail in appendices.

### 3.1. Dichotomous time-varying covariate with at most one change from untreated to treated

Let *t*_0_ denote the time at which the time-varying covariate changes from unexposed (*Z* = 0) to exposed (*Z* = 1). Thus, *z*(*t*) = 0 for *t* < *t*_0_, while *z*(*t*) = 1 for *t ≥ t*_0_. We determine the cumulative hazard function for *t* < *t*_0_ and for *t ≥ t*_0_, because the cumulative hazard function will have different functional form over these two domains. Therefore, the definition of the inverse of the cumulative hazard function will have a piece-wise definition.

#### 3.1.1. Exponential distribution of event times

If event times follow an exponential distribution, one can simulate survival time as


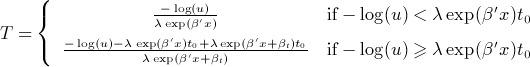
(1)

where *u* ∼ *U*(0,1). The derivation of this expression is presented in Appendix A.

#### 3.1.2. Weibull distribution of event times

If event times follow a Weibull distribution, then, if *t* < *t*_0_, the cumulative hazard function is equal to


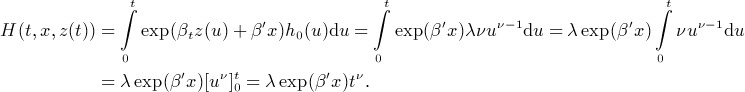


If *t ≥ t*_0_, then the cumulative hazard function is equal to


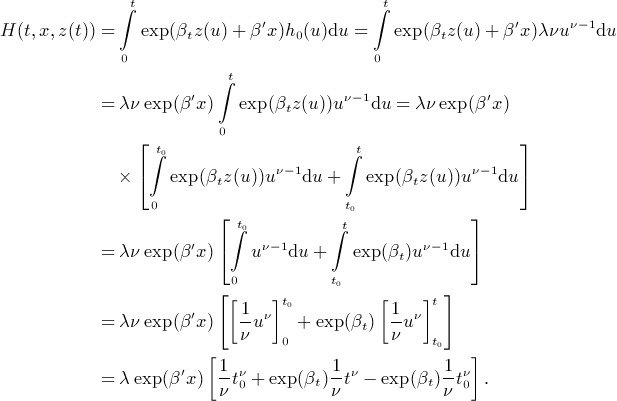


Therefore, we have that 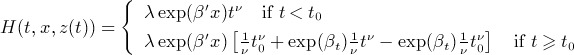.

The domain of the cumulative hazard function can be partitioned into two intervals: *D*_1_ = (0, *t*_0_) and *D*_2_ = [ *t*_0_, ∞ ). Let 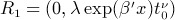 and 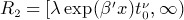 denote the range of the cumulative distribution function associated with the domains *D*_1_ and *D*_2_, respectively. We determine the inverse of the cumulative hazard function for values in *R*_1_ and *R*_2_ separately.

The inverse of the cumulative hazard function when 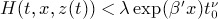 is given by: *H*(*t*,*x*,*z*(*t*)) = *λ*exp(*β* ′ *x*)*tν*, thus (*H*(*t*,*x*,*z*(*t*)) / *λ*exp(*β* ′ *x*)) = *tν*. Thus, *t* = (*H*(*t*,*x*,*z*(*t*)) / *λ*exp(*β* ′ *x*)) ^1 / *ν*^. Therefore, we have that


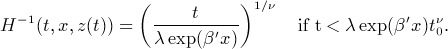


The inverse of the cumulative hazard function when 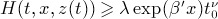 is given by: 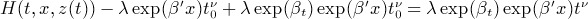. Then


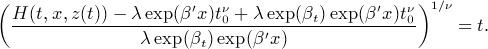


The inverse of the cumulative hazard function is 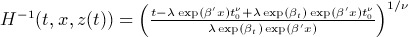 when 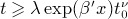.

Therefore, we can simulate a survival time as


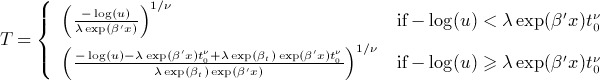
(2)

where *u* ∼ *U*(0,1).

#### 3.1.3. Gompertz distribution of event times

If event times follow a Gompertz distribution, one can simulate a survival time as


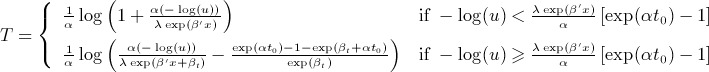
(3)

where *u* ∼ *U*(0,1). The derivation of this expression is presented in Appendix B.

### 3.2. Continuous time-varying covariate

In this section, we assume that the time-varying covariate *z*(*t*) is proportional to *t*: *z*(*t*) = *kt*, with *k* > 0. This would be the case when a subject is exposed to a uniform dose during each unit of time during follow-up. Examples include subjects who take a certain dose of medication each day, or workers who are exposed to a fixed dose of radiation each day. In this section, we do not require a piece-wise definition of the cumulative hazard function. Thus, we can proceed more simply than in Section 3.1.

#### 3.2.1. Exponential distribution of event times

If survival times follow an exponential distribution, an event time can be generated as


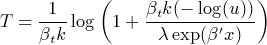
(4)

where *u* ∼ *U*(0,1). The full derivation of this expression is reported in Appendix C.

#### 3.2.2. Weibull distribution of event times

If survival times follow a Weibull distribution, we have that


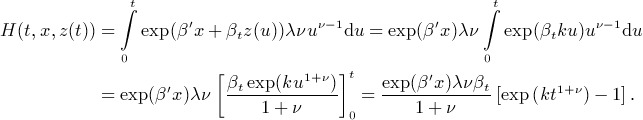


Therefore, we have that


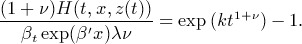


Then 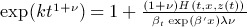; 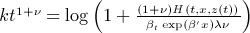; 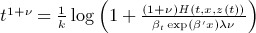; and 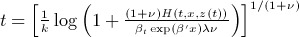.

Therefore, we have that 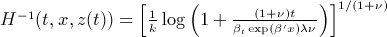.

One can then simulate a survival time as


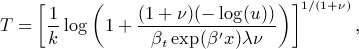
(5)

where *u* ∼ *U*(0,1).

#### 3.2.3. Gompertz distribution of event times

If event times follow a Gompertz distribution, one can simulate a survival time as


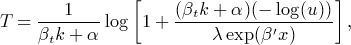
(6)

where *u* ∼ *U*(0,1). The full derivation of this expression is presented in Appendix D.

### 3.3. Dichotomous time-varying covariate with multiple changes between treated and untreated

In this section, we consider the setting in which a subject may repeatedly move between untreated and treated conditions. For the purposes of these derivations we will assume that all subjects are untreated at *t* = 0. Let *t*_1_ denote the time at which the binary time-varying covariate changes from unexposed (*Z* = 0) to exposed (*Z* = 1); let *t*_2_ denote the time at which the binary time-varying covariate changes from exposed (*Z* = 1) to unexposed (*Z* = 0); finally, let *t*_3_ denote the time at which the time-varying covariate changes from unexposed (*Z* = 0) to exposed (*Z* = 1). In these derivations, we assume that a subject experienced three switches in treatment status (at times *t*_1_, *t*_2_, and *t*_3_). The subsequent derivations can readily be modified to accommodate a different number of changes in treatment status. We only present the derivations for the setting in which event times follow a Weibull distribution, because all the derivations will be similar to those in Section 3.1.

If event times follow a Weibull distribution with scale parameter *λ* and shape parameter *ν*, then, using methods similar to those above, the cumulative hazard function is equal to


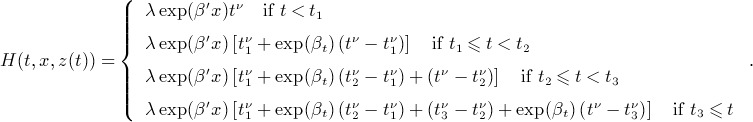


The domain of the cumulative hazard function can be divided into four mutually exclusive intervals *D*_1_ = (0, *t*_1_), *D*_2_ = [ *t*_1_, *t*_2_), *D*_3_ = [ *t*_2_, *t*_3_), *D*_4_ = [ *t*_3_, ∞ ). The range of cumulative hazard function over each of these intervals is


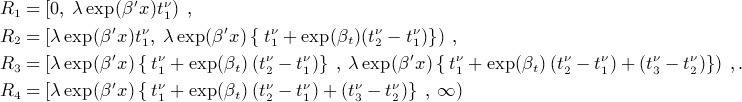


By inverting each of the piece-wise components of the cumulative hazard function, we have


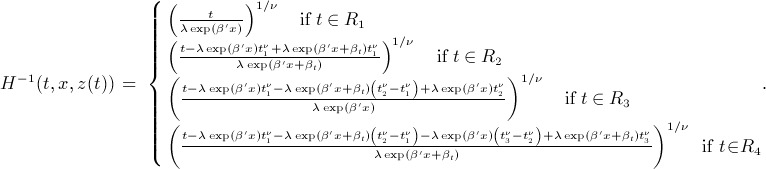


One can therefore simulate a survival time as *H*^− 1^( − log(*u*),*x*,*z*), where the value of − log(*u*) determines which of the four component functions for the inverse of the cumulative hazard function is used.

### 3.4. Comparison of generating survival times with time-varying covariates with generating survival times with time-invariant covariates

In this section, we briefly compare our results with prior work on generating survival times for the Cox model with time-invariant covariates. In Table I, we described the formulas for generating survival times with time-invariant covariates (based on the prior work of Bender *et al.* [2]). We compare these with our formulas for simulating event times with a binary time-varying covariate that changes at most once from untreated to treated (Section 3.1). In our derivations, if one fixes *t*_0_ to be equal to 0 (i.e., treatment occurs only at time zero, and then remains fixed during the duration of follow-up), then each formula for generating survival times reduces to the comparable formula from Bender *et al.* in Table I. Similarly, if the binary covariate is time-varying, but if the effect of that covariate is zero (*β*_*t*_ = 0), then each expression reduces to the comparable formula in the Table I.

## 4. Application — power calculation for Cox regression model with a time-varying covariate

We illustrate the utility of our data-generating processes by using Monte Carlo simulations to estimate the statistical power to detect as statistically significant the hazard ratio associated with different dichotomous time-varying covariates. So that our simulations would reflect a realistic setting, we used data from the Ontario Myocardial Infarction Database (OMID), a population-based database of patients hospitalized with an acute myocardial infarction (AMI or heart attack) created by linking electronic administrative health care databases in the Canadian province of Ontario. The creation of the OMID database is described in greater detail elsewhere [9]. For this illustration, we selected the 115,856 patients hospitalized with an AMI in Ontario between 2000 and 2005. Each patient was followed until his or her death, with subjects being censored on March 31, 2006. Thirty-two percent of the patients had died by March 31, 2006, while the remaining 68% were still alive on this date.

Our objective was to determine the statistical power to detect as statistically significant the effect of treatment after adjusting for the predictors of mortality contained in the Ontario AMI mortality prediction rule: age, gender, measures of cardiac severity (congestive heart failure, cardiogenic shock, arrhythmia, and pulmonary edema), and comorbid status (diabetes mellitus with complications, stroke, acute and chronic renal disease, and malignancy). The nine measures of cardiac severity and comorbid conditions were derived from the ICD-9/10 codes present in the secondary diagnostic fields of the hospitalization database. The derivation and validation of the prediction rule is described elsewhere [10].

We estimated the statistical power to detect as statistically significant the regression coefficient associated with a dichotomous treatment in a sample of 2500 subjects whose covariates were similar to that of the Ontario AMI population. We used a Cox proportional hazards regression model to regress time-to-death on age, sex, and the nine risk factors that comprise the Ontario AMI mortality prediction model in the sample of 115,856 AMI patients. For each of the 115,856 subjects, we determined the linear predictor (*β*^*t*^*X*) from the fitted regression model. We then chose a sample of size 2500 from the overall population of 115,856 using random sampling with replacement. We considered five different types of binary treatments: first, 50% of the subjects were assigned to a fixed, time-invariant treatment at baseline. The remaining subjects were untreated for the duration of follow-up. Second, all subjects were assigned to receive a treatment. The time of receipt of treatment was generated for each subject from a *U*(1,13581) distribution (i.e., each subject changed from untreated to treated on a day chosen at random between 1 and 13,581). Once treatment was received (at the randomly generated time), subjects remained treated or exposed for the duration of follow-up. Note that in this setting, subjects could die prior to the time that treatment was to be assigned. The third scenario was similar to the second, with the sole difference being that a *U*(1,6000) distribution was used to generate times at which treatments were assigned. Fourth, 50% of subjects were assigned to receive a time-varying treatment. For those subjects assigned to receive the treatment, there were three switches between treatment status. Subjects switched from untreated to treated at time (1/3) * 13,581, from treated to untreated at time (2/3) * 13,581, and from untreated to treated at time 13,581. The fifth scenario was similar to the fourth, except that there were only two switches between treatment status. Subjects switched from untreated to treated at time (1/3) * 13,581 and from treated to untreated at time equal to (2/3) * 13,581.

For each of the five scenarios, we randomly generated event times using the linear predictor (*β*^*t*^*X*), the time at which treatment status was changed from untreated to treated using a Cox–Weibull model with shape and scale parameters of 0.6 and 0.001, respectively. We allowed the hazard ratio for the effect of treatment to vary from 0.5 to 0.95 in increments of 0.05. We considered two different censoring mechanisms. First, event times were censored at the 32 ^nd^ percentile of event-times so that, as in the OMID database, events would only be observed to occur for 32% of the subjects, with the remaining 68% of subjects being censored. Using this censoring mechanism there was a fixed time at which all subjects were censored. Second, for each subject, we determined a random censoring time so that censoring times were uniformly distributed between one and the 75 ^th^ percentile of event times. Each subject's survival time was the lower of the event time and the censoring time. This second approach used a random censoring time.

In the sample of 2500 subjects with simulated outcomes, we used a Cox model to regress survival time on treatment status (as a time-varying covariate), age, sex, and the nine variables in the AMI mortality risk model. We noted whether the regression coefficient associated with treatment status was statistically significant (*P ≤*0.05). This process was repeated 1000 times, and the proportion of samples in which the treatment effect was statistically significant was determined.

**Figure 1:**
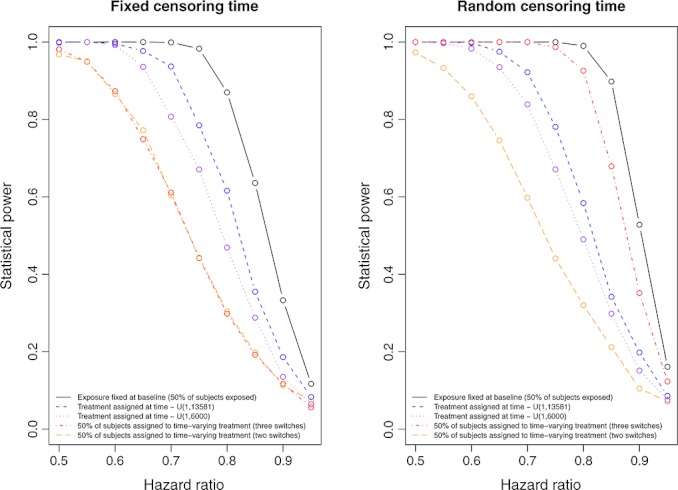
Statistical power to detect a binary treatment as statistically significant.

In the second scenario under the first censoring mechanism, the percentage of subjects who died prior to receipt of treatment varied from 23% to 28%, while in the third scenario this percentage was approximately 16%. The statistical power to detect as statistically significant the different forms of binary treatments under the first censoring mechanism are described in the left panel of Figure 1. The highest statistical power was observed when 50% of the subjects were assigned to a time-invariant treatment at baseline and the remaining subjects were untreated over the duration of follow-up. The two scenarios in which time-to-treatment was randomly determined from a uniform distribution had intermediate levels of statistical power. The two scenarios in which half of the subjects were assigned to a treatment that involved alternating between untreated and treated had the lowest statistical power. With one exception, comparable findings were observed under a random censoring mechanism (right panel of Figure 1). The single exception was that with a random censoring mechanism, the power to detect as statistically significant a treatment that involved three switches approached that of a time-invariant treatment that was fixed at baseline.

## 5. Discussion

We described data-generating processes for the Cox proportional hazards model with time-varying covariates when event times follow an exponential, Weibull, or Gompertz distribution. We considered three types of time-varying covariates: first, a dichotomous time-varying covariate that can change at most once from untreated to treated (e.g., organ transplant); second, a continuous time-varying covariate such as cumulative exposure to a constant dose of radiation or to a pharmaceutical agent when the medication is used for a chronic condition; third, a dichotomous time-varying covariate with a subject being able to move repeatedly between treatment states. In each setting, we derived closed-form expressions that allow one to simulate event times so that event times are related to a vector of time-invariant covariates and to a single time-varying covariate. Formulas 1 to 6 are the closed-form expressions for the first two types of time-varying covariates for exponential, Weibull, and Gompertz distributions.

Several prior studies have developed methods for simulating event time data for the Cox proportional hazards model. As noted in Section 2, both Leemis and Bender *et al.* described methods to generate survival times for a Cox proportional hazards model with time-invariant covariates [1, 2]. Mackenzie and Abrahamowicz described methods for simulating survival data that allowed for time-dependence of the hazard ratio and that allow one to specify the marginal distribution of event times and covariate distributions [11]. Finally, Beyersmann *et al.* described methods to simulate competing risks data in survival data [12].

A small number of prior papers have examined data-generating processes for survival times with time-varying covariates. Leemis *et al.* briefly described methods based on inverting the cumulative hazard function to generate survival times in settings with time-varying covariates [13]. The current study builds and extends this prior work of Leemis *et al.*, who did not use the logarithmic link function for relating the hazard function to the linear predictor. All of our derivations were based on the logarithmic link function, because this is the link function that is almost universally used with the Cox proportional hazard regression model in the biomedical sciences. Furthermore, the derivations by Leemis *et al.* involve a single time-varying covariate and did not incorporate time-invariant covariates. Finally, Sylvestre and Abrahamowicz examined two different algorithms for simulating event times conditional on time-varying covariates [14]. The first algorithm was based on a permutational algorithm, while the second was based on a binomial model. They considered a modification of the first to incorporate a rejection sampler. An advantage to the methods described in the current paper is that we have presented closed-form expressions for simulating event times. This should result in greater efficiency in Monte Carlo simulations compared with methods based on the algorithms used in the prior paper. Sylvestre and Abrahamowicz noted that the permutational algorithm will be computationally intensive when the number of events need to be generated is large [14], p. 2621. In contrast, our closed form expressions should be relatively insensitive to the number of events that need to be generated.

We illustrated the utility of our data-generating processes by estimating the statistical power to detect as statistically significant a time-varying treatment after adjusting for a set of fixed or time-invariant covariates. We found that the statistical power to detect a non-null hazard ratio when the treatment or exposure was time-varying was lower than the power to detect a non-null hazard ratio when the treatment was fixed at baseline. The use of the described data-generating processes will allow biostatistical investigators to estimate statistical power and select appropriate sample sizes in complex settings in which there are both time-invariant and time-varying covariates.

Statistical simulations are playing an increasingly important role in modern statistical research. They allow the investigation of performance and properties of estimators and models in settings in which analytic calculations are either very difficult or not tractable. Given the ubiquitous use of the Cox proportional hazards model in biomedical research and the frequency with which time-varying covariates occur in medical research, the data-generating processes described in the current paper will be of use to statisticians examining different properties of the Cox regression model.

## Appendix A. Dichotomous time-varying covariate with at most one change from untreated to treated: exponential distribution of event times

If event times follow an exponential distribution, then, if *t* < *t*_0_, the cumulative hazard function is equal to

If *t ≥ t*_0_, then the cumulative hazard function is equal to

Therefore, we have that .

The domain of the cumulative hazard function ((0, ∞ )) can be partitioned into two mutually exclusive intervals: *D*_1_ = (0, *t*_0_) and *D*_2_ = [ *t*_0_, ∞ ). The range of the cumulative hazard function over each of *D*_1_ and *D*_2_ are *R*_1_ = (0, *λ*exp(*β* ′ *x*)*t*_0_) and *R*_2_ = [ *λ*exp(*β* ′ *x*)*t*_0_, ∞ ), respectively. We will determine the inverse of the cumulative hazard function for values in *R*_1_ and *R*_2_, separately.

The inverse of the cumulative hazard function when *H*(*t*,*x*,*z*(*t*)) < *λ*exp(*β* ′ *x*)*t*_0_ is given by: *H*(*t*,*x*,*z*(*t*)) = *λ*exp(*β* ′ *x*)*t*; thus *t* = *H*(*t*,*x*,*z*(*t*)) / *λ*exp(*β* ′ *x*). Therefore, *H*^− 1^(*t*,*x*,*z*(*t*)) = *t* / *λ*exp(*β* ′ *x*) if *t* < *λ*exp(*β* ′ *x*)*t*_0_.

The inverse of the cumulative hazard function when *H*(*t*,*x*,*z*(*t*)) *≥ λ*exp(*β* ′ *x*)*t*_0_ is given by

Therefore, the inverse of the cumulative hazard function is

Thus, we can simulate a survival time as

## Appendix B. Dichotomous time-varying covariate with at most one change from untreated to treated: Gompertz distribution of event times

If event times follow a Gompertz distribution, then, if *t* < *t*_0_, the cumulative hazard function is equal to

If *t ≥ t*_0_, then the cumulative hazard function is equal to

Therefore, we have that

As with the exponential and Weibull distribution, we will determine the inverse of the cumulative hazard function for values above and below the threshold value .

The inverse of the cumulative hazard function when  is given by: . Thus, ;  ; and . Thus,  if .

The inverse of the cumulative hazard function when  is given by:  if .

Thus, we can simulate a survival time as

## Appendix C. Continuous time-varying covariate: exponential distribution of event times

If survival times follow an exponential distribution, we have that

Consequently, we that the inverse cumulative hazard function is

Therefore, an event time can be generated as

where *u* ∼ *U*(0,1).

## Appendix D. Continuous time-varying covariate: Gompertz distribution of event times

If survival times follow a Gompertz distribution, we have that

Thus, we have that ;

Then ; ; and .

Therefore, the inverse of the cumulative hazard function is

Then, one simulate a survival time as

where *u* ∼ *U*(0,1).
